# A Mantis-Inspired Multi-Quadrupole Adaptive Landing Gear Design and Performance Study

**DOI:** 10.3390/biomimetics10050327

**Published:** 2025-05-17

**Authors:** Yichen Chu, Zhifeng Lv, Shuo Gu, Yida Wang, Tianbiao Yu

**Affiliations:** 1 School of Mechanical Engineering and Automation, Northeastern University, Shenyang 110819, China; 2310088@stu.neu.edu.cn (Y.C.); 20224811@stu.neu.edu.cn (Z.L.); tbyu@me.neu.edu.cn (T.Y.); 2 Beijing Lindong Bionic Science and Technology Limited Company, Beijing 100080, China; 3 Student Guidance Service Center, Northeastern University, Shenyang 110819, China; 4 School of Information Science and Engineering, Northeastern University, Shenyang 110819, China; 2400756@stu.neu.edu.cn

**Keywords:** bionics, landing gear, institutional design, cushioning and damping absorption, energy recovery

## Abstract

This paper investigates and designs an adaptive landing gear inspired by the passive adaptation mechanism of the praying mantis on intricate landing surfaces to improve the landing safety of unmanned aerial vehicles (UAVs) in complicated terrain situations. A new passive adaptation structure utilizing multiple mutually perpendicular four-bar mechanisms is developed to address the limitations of the typical fixed truss structure landing gear. The system employs a singular laser range sensor locking mechanism, thereby significantly diminishing the control and structural complexity. The design incorporates a parallelogram mechanism to achieve the adaptation of different height differences through the mechanism’s deformation. The buffer damping mechanism and locking mechanism are engineered to augment the safety of the landing process and enhance the energy recovery rate. The circuit design employs the STC32G and Keil C251 microcontroller for development, thus achieving the automatic control of the landing gear. The experimental results demonstrate that the adaptive landing gear suggested in this paper can successfully adjust to the complex landing surface and has a good energy recovery performance. This aids in the advancement of UAVs in the field of complex environment applications and offers a safe, dependable, and creative solution for UAV landing scenarios in complex terrains.

## 1. Introduction

During the advancement of science and technology, unmanned aerial vehicle (UAV) technology, due to its distinctive advantages, has exhibited a significant application potential across various domains. Rotary-wing UAVs with vertical takeoff [1] and landing capabilities are essential in difficult scenarios, including field exploration missions, border patrols, and emergency rescues, because of their superior maneuverability and adaptation [2,3,4]. Landing gear, a critical component of the UAV, is vital to the safety and stability of UAV takeoff and landing operations [5]. Conventional UAV landing gears predominantly consist of fixed truss frameworks [6,7]. This structure can fulfill usage needs under relatively simple flight conditions to a certain extent. Nonetheless, when confronted with landing operations in intricate terrains or metropolitan environments devoid of aprons, the structure exhibits significant constraints, hence severely restricting the application range and operational effectiveness of UAVs [8,9]. Consequently, the investigation and development of large-scale UAV landing gears capable of adapting to intricate landing surfaces and ensuring stable landings have emerged as a significant research focus in the UAV domain today.

The United States Defense Advanced Research Projects Agency (DARPA) initially proposed the adaptive landing mission adaptive rotor (MAR) project [10], which employed a legged construction to facilitate the vehicle’s adaptation to various terrains during takeoff and landing operations. Subsequent research on adaptable landing gear concentrated on legged adaptive landing gear [11,12]. For instance, Hongyan Tang et al. [13] at Fudan University developed a three-legged landing gear featuring a slider linkage mechanism that accommodates varying landing heights by adjusting the slider’s position via a screw linked to a motor. Yuri S. Sarkisov et al. [14] at the Skolkovo Institute of Science and Technology created an adaptive four-legged landing gear, which adjusts to intricate landing surfaces through two motors for each leg and torque sensors at the knee joints. Boris Stolz et al. [15] at ETH Zurich engineered an adaptive four-legged landing gear, where the motors are supplemented by a parallel configuration of motors and brakes to mitigate the load impacts during hard landings. Jia Ren et al. [16] from the AVIC Aircraft Strength Research Institute developed a bionic landing gear featuring six legs, employing two crank rocker mechanisms to accommodate varying landing point elevations. Paul M. Nadan and Christopher L. Lee [17] from the Franklin W. Olin College of Engineering created a bionic, avian-inspired, two-legged landing gear, enabling foot functionality in intricate environments via two cable-driven, multi-segmented landings. The research team at the Harbin Institute of Technology [18] optimized the motion control strategy of the legs through a dynamic analysis of the multilegged landing gear, enhancing its adaptability to complex terrains.

In their investigation of the landing gear control strategy, Arık, A.E., and Boğaç Bilgiç [19] proposed a fuzzy control system utilizing a grease pneumatic damper that was presented to efficiently mitigate vibrations caused by landing impacts during descent. The landing performance was demonstrated to be good across different landing speed situations, exhibiting strong robustness. Quang-Ngoc Le et al. [20] formulated a mathematical model for the dynamics of landing gear during landing and devised two control modes, Model Predictive Control (MPC) and intelligent control, which demonstrated the enhanced damper efficiency and greater adaptability of the landing gear system across various landing situations. Quoc-Viet Luong et al. [21] developed an intelligent controller for aircraft landing gear magnetorheological dampers utilizing supervised neural network control, enhancing the damper efficiency by 6% without precise data by creating a landing shock test environment on the dSPACE platform, using hybrid controller data as the supervised learning target. In the field of landing gear material applications, Yen-Chu Liang et al. [22] developed a landing gear utilizing various orientations of carbon fiber-reinforced polymer (CFRP) composites, resulting in reduced weight and improved strength characteristics. Camil Lancea et al. [23] utilized the fused filament fabrication (FFF) technique to create a model of UAV composite landing gear, also discovering that a high-quality carbon fiber-reinforced polymer (CFRP) exhibited superior mechanical properties in the fabrication of the landing gear. However, the FFF process encountered defects, including voids, deposition issues, and the micro-cracking of the material during fabrication.

Nonetheless, a comprehensive examination of the current findings indicates that existing adaptive landing gears predominantly utilize active or semi-active adaptation mechanisms, which typically depend on numerous sensors and the control system working in collaboration [24]. The landing gear adjusts to the intricate terrain by precisely measuring the ground environment and dynamically modifying the length of each landing leg according to the measurement outcomes [25]. This technique, while capable of fulfilling landing criteria in complex surroundings to some degree, inherently presents numerous issues, including a complicated structure, challenging control, elevated costs, and various practical application difficulties. The utilization of numerous sensors elevates the system’s power consumption and the likelihood of failure [26]. Furthermore, the intricate control algorithms necessitate an exceptionally high processor performance and are vulnerable to interference during signal transmission, thereby impacting the stability of the adaptation effect [27]. Simultaneously, the elevated expense constrains its widespread utilization.

This paper addresses the problem by drawing inspiration from the passive buffer adaptation mechanism observed in the landing process of the praying mantis and proposes an innovative landing gear utilizing multiple perpendicular four-bar mechanisms for a passive adaptation to ground obstacles. The rod set is equipped with a locking mechanism and a single laser distance sensor to effectively adapt to complicated obstacle surfaces, significantly diminishing control difficulty and structural complexity. This paper initially conducts the mechanism design of passive adaptive landing gear utilizing multiple four-bar mechanisms, followed by design calculations for the adaptive range of the mechanism and the installation position of the shock absorber through trigonometric functions. Subsequently, drawing inspiration from bicycle disk brakes [28], a wire-controlled disk brake locking mechanism is incorporated into each variable four-bar mechanism to enhance the safety of the landing gear’s overall mechanism by locking. The trapezoidal structural design and the pre-compression of the shock absorber spring enhance landing safety and the energy recovery rate on difficult landing surfaces. This study selects the STC32G as the primary control chip, with Keil C251 for microcontroller development. Testing in a simulated complicated environment confirms the viability of the landing gear for adaptive landing.

## 2. Design and Analysis

### 2.1. Landing Gear Mechanism Design

#### 2.1.1. Mechanism Design

During the design phase of the landing gear mechanism, it is observed that the parallelogram mechanism with equal opposite sides has an unconstrained deformable angle relative to the other four-bar mechanisms, resulting in a significantly larger adaptable angle [29]. Since the four sides of the parallelogram mechanism are consistently parallel in pairs, the phase of the main body can be adjusted by the phase of the end legs to achieve phase correlation. This study utilizes the parallelogram mechanism as the foundation for the adjustable rod assembly in the overall mechanism design. The parallelogram mechanism, being a planar four-bar system, can only adjust the angle within a single plane. However, the uneven road surface where the landing gear makes contact may have various barriers. This paper further draws inspiration from the mantis’s passive buffer adaptation system when it is landing [30].

**Figure 1:**
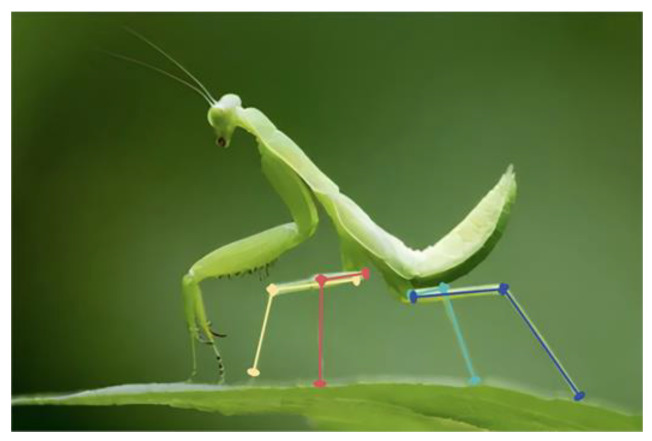
Mantis landing leg adaptation schematic.

**Figure 2:**
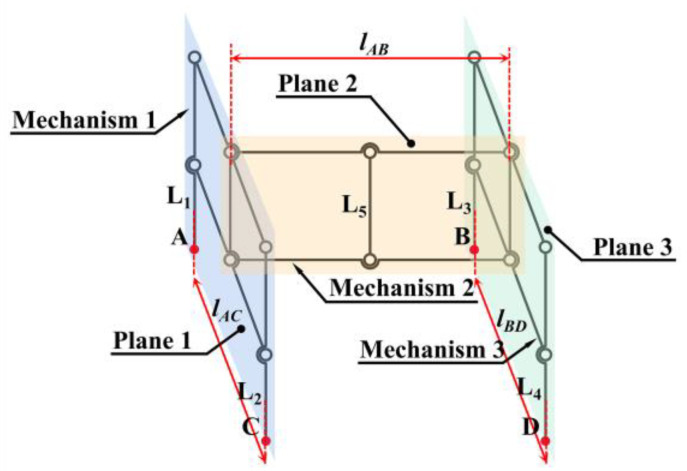
A sketch of the overall landing gear mechanism. Where plane 1 is vertical to plane 2, and plane 1 is parallel to plane 3. There exists *L*_AD_ = *L*_BD_ = *L*_xl_ for mechanisms 1 and 3 while maintaining a rectangular state. *L*_AB_ = *L*_CD_ = *L*_yl_, *L*_1_ = *L*_2_ = *L*_3_ = *L*_4_ = *L*_zl_, *L*_5_ = 1/2 *L*_zl_ for mechanism 2 while maintaining a rectangular state.

As shown in Figure 1, after completing the landing, the direction of the mantis’s tibia is maintained at a certain angle to the direction of gravity and inclined towards the position of the body’s center of gravity to ensure the stability of the main body. Inspired by its landing attitude, the three parallelogram mechanisms are spatially combined in this article, with mechanism 1 and mechanism 3 in parallel planes 1 and 3, respectively, and coupled by mechanism 2 in vertical plane 2, as shown in Figure 2. Mechanisms 1 and 3 are connected at the midpoint by mechanism 2. The entire mechanism makes contact with the ground at four points: *A*, *B*, *C*, and *D*. The overall system comprises three parallelogram mechanisms, ensuring that the five rods, *L*_1_, *L*_2_, *L*_3_, *L*_4_, and *L*_5_, remain parallel. Fix the vehicle with the *L*_5_ rod. During the descent of the vehicle, ensure that via its lift, the orientation of the *L*_5_ rod is parallel to the gravitational vector, thereby aligning the four rods, *L*_1_, *L*_2_, *L*_3_, and *L*_4_, with the direction of gravity to preserve the stability of the vehicle’s main body connected to the *L*_5_ rod.

Three components work together to enable the entire mechanism to adapt to the complicated obstacle surface:

1. Mechanism 1 is used to adapt the height difference between two places, A and C, in plane 1.
2. Mechanism 3 is used to adapt the height difference between places B and D in plane 3.
3. Mechanism 2 is used to adapt the height difference between the median height values of mechanisms 1 and 3.

**Figure 3:**
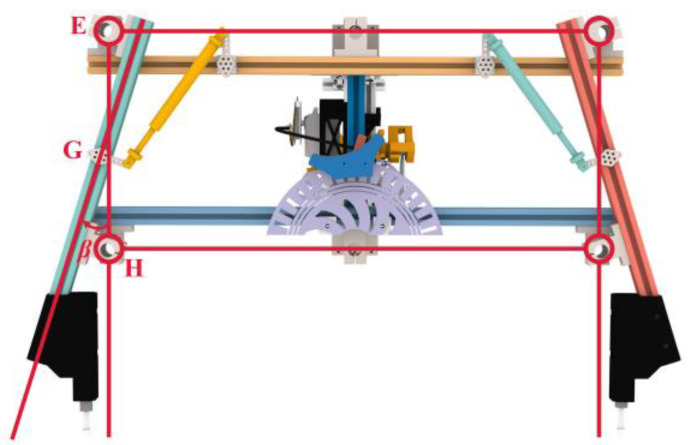
Improved landing gear overall structure.

The overall mechanism adopts the parallelogram mechanism as the theoretical member. Nevertheless, the support rods that are perpendicular to the landing surface in the theoretical components are susceptible to tilting due to the unequal forces exerted on their inner and outer sides during the landing process. Motivated by the mantis’s posture, where the tibia converges towards the body’s center of gravity upon landing, the design of the adaptive landing gear is enhanced. As shown in Figure 3, the theoretical components of the mechanism remain as parallelograms. By extending inward at revolute pair *E* and expanding outward at revolute pair *H*, the supporting rod is given an outward inclination angle *β*. It extends inward at hinge *E* and outward at hinge *H* to ensure that hinge *G* always lies on the line connecting *E* and *H*. The theoretical member’s structural configuration is optimized to a trapezoidal form, featuring a narrow top and a broad base, which enhances its stability during landing.

#### 2.1.2. Adaptation Range Calculation

**Figure 4:**
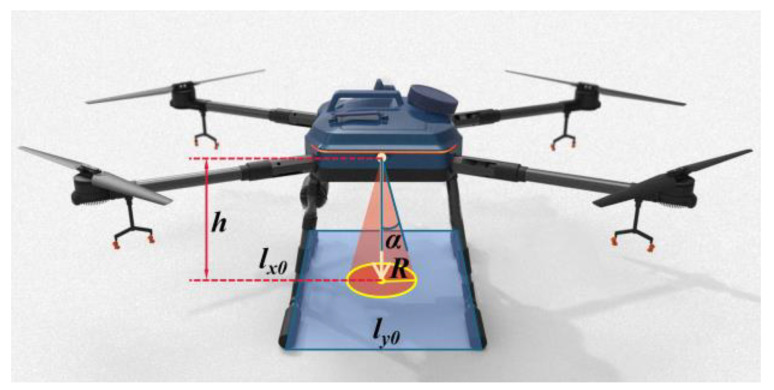
The range of the center of gravity shift during the rigid landing gear motion.

The landing gear designed in this paper is a four-bar mechanism with equal and opposite sides, which can compensate for height differences. Nevertheless, due to the extensive deformable range of the parallelogram mechanism, it is necessary to set the range of the mechanism’s movement angle. The range of motion angles of the passive adaptive landing gear mechanism can be set by adjusting the safety coefficient of the support surface of the multi-quadruple rod passive adaptive landing gear, which is greater than that of the conventional rigid landing gear. Initially, the conventional rigid landing gear is examined, as illustrated in Figure 4. The height of the center of gravity above the ground post-UAV landing is denoted as *h*; the angle between gravitational force and lift during the UAV takeoff and landing is represented by *α*; the length of the UAV landing gear skid is *l*_x0_; and the distance between the two skids is *l*_y0_.

Shown in Figure 4, the landing gear of the UAV:

(1) R=h⋅tanα

(2) $$S^{\prime }=\pi R^{2}=\pi {\left(h\cdot \tan\alpha \right)}^{2}$$

(3) $$[S]=4R^{2}$$

(4) $$S=l_{x0}\cdot l_{y0}$$

where *R* is the center of gravity offset projection radius, *S*′ is the center of gravity offset projection area, [*S*] is the minimum safe area of the UAV landing gear, and *S* is the landing gear support area of the UAV. Then, the safety factor *n*_L0_ of the landing gear support surface is

(5) $$n_{L0}=\frac{S}{\left[S\right]}$$

**Figure 5:**
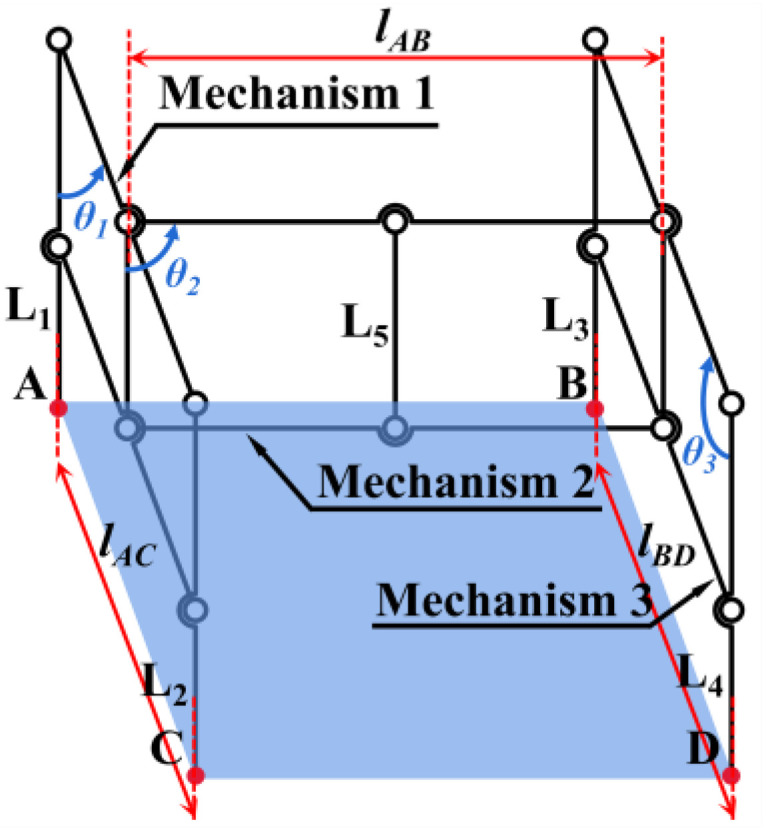
A schematic diagram of the deformation angle of the mechanism.

Wibawa [31] calculated the minimum safety factor *n*_L0_ for rigid landing gear with rounded corners to be about 2.97 by the finite element method. This paper presents an innovatively designed, multi-four-bar, passive adaptive landing gear that adjusts to the ground height upon landing on complex surfaces, with mechanisms 1, 2, and 3 each contributing to the deformation required for this adaptation. As shown in Figure 5, the deformation angles produced by the three mechanisms are *θ*_1_, *θ*_2_, and *θ*_3_, respectively. After deformation:

(6) $$\left\{\begin{array}{l} l_{AC}=l_{x1}\cdot \sin\theta_{1}\\ l_{BD}=l_{x1}\cdot \sin\theta_{3}\\ l_{AB}=l_{y1}\cdot \sin\theta_{2} \end{array}\right.$$

(7) $$S_{d}=l_{AB}\left[\left(l_{AC}+l_{BD}\right)/2\right]$$

(8) $$\left\{\begin{array}{l} \Delta y_{AC}=y_{A}-y_{C}=l_{x1}\cdot \cos\theta_{1}\\ \Delta y_{DB}=y_{D}-y_{B}=l_{x1}\cdot \cos\theta_{3}\\ \Delta y_{AB}=y_{A}-y_{B}=l_{y1}\cdot \cos\theta_{2}+\left(l_{x1}/2\right)\cdot \cos\theta_{1}-\left(l_{x1}/2\right)\cdot \cos\theta_{3}\\ \Delta y_{CD}=y_{C}-y_{D}=l_{y1}\cdot \cos\theta_{2}-\left(l_{x1}/2\right)\cdot \cos\theta_{1}+\left(l_{x1}/2\right)\cdot \cos\theta_{3}\\ \Delta y_{AD}=y_{A}-y_{D}=l_{y1}\cdot \cos\theta_{2}+\left(l_{x1}/2\right)\cdot \cos\theta_{1}+\left(l_{x1}/2\right)\cdot \cos\theta_{3}\\ \Delta y_{BC}=y_{B}-y_{C}=l_{y1}\cdot \cos\theta_{2}-\left(l_{x1}/2\right)\cdot \cos\theta_{1}-\left(l_{x1}/2\right)\cdot \cos\theta_{3} \end{array}\right.$$

where *S*_d_ is the horizontal projection of the support area of the landing gear after the deformation of the overall mechanism. To make the maximum adaptable height difference of the neighboring points in the four points *A*, *B*, *C*, and *D* the same:

(9) $$\Delta y_{AC}=\Delta y_{DB}=\Delta y_{AB}=\Delta y_{CD}$$

(10) $$\left\{\begin{array}{l} \theta_{1\max}=\theta_{3\max}\\ \cos\theta_{1\max}=l_{\mathrm{yl}}\cos\theta_{2\max}/l_{x1} \end{array}\right.$$

where *θ*_1max_, *θ*_2max_, and *θ*_3max_ are the maximum values of the movement angles of mechanism 1, mechanism 2, and mechanism 3, respectively. The overall mechanism has axisymmetric and centrosymmetric features.

(11) $${S_{d}}^{\prime }=l_{y1}\sin\theta_{2\max}\left[\left(l_{x1}\sin\theta_{1\max}+l_{x1}\sin\theta_{3\max}\right)/2\right]$$

where *S*_d_′ denotes the horizontal projection of the landing gear’s support area once the mechanism attains the maximum deformation angle, representing the minimal support area. To ensure that the safety of the adaptive landing gear is not inferior to that of the rigid landing gear, the following conditions must be met:

(12) $$\left\{\begin{array}{l} {S_{d}}^{\prime }\geq S\\ l_{y1}\sin\theta_{2\max}\geq l_{y0}\\ \left(l_{x1}\sin\theta_{1\max}+l_{x1}\sin\theta_{3\max}\right)/2\geq l_{x0} \end{array}\right.$$

(13) $${n_{L}}^{\prime }=\frac{{S_{d}}^{\prime }}{\left[S\right]}$$

(14) $$\left\{\begin{array}{l} S_{d1}=l_{y1}\sin\theta_{2}\left[\left(l_{x1}\sin\theta_{1}+l_{x1}\sin\theta_{3}\right)/2\right]\\ n_{L1}=\frac{S_{d1}}{\left[S\right]} \end{array}\right.$$

where *n*_L_′ is the support surface safety coefficient of the adaptive landing gear after the mechanism reaches the maximal deformation angle. The mechanism support surface safety coefficient is denoted by *n*_L1_. The landing gear safety factor *n*_L1_ can be estimated to be approximately 3.28 using the landing gear data.

**Figure 6:**
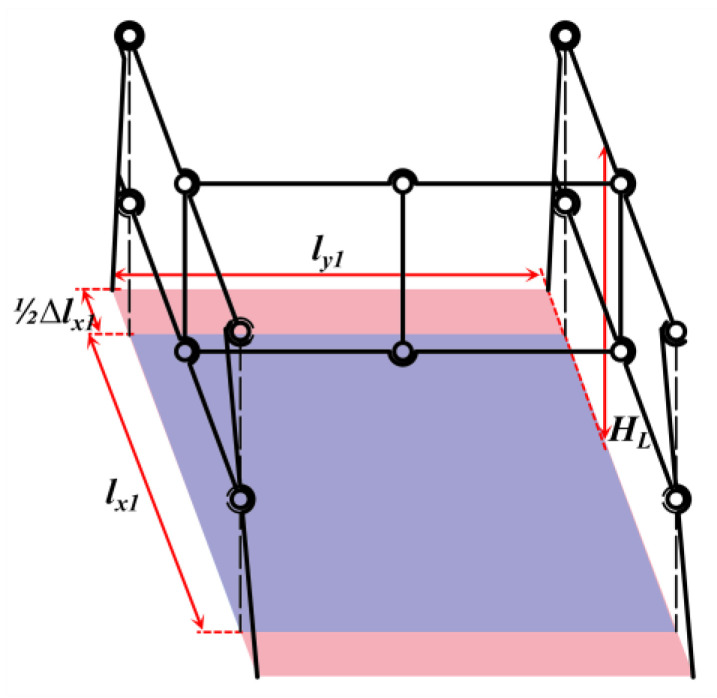
A schematic diagram of the support surface after the tilting of the support member.

Due to the final design of the landing gear, support bars are tilted outward at an angle of *β*, the support area of the adaptive landing gear is also varied, as shown in Figure 6:

(15) $$\frac{1}{2}\Delta l_{x1}=h_{L}\cdot \tan\beta$$

(16) $$\left\{\begin{array}{l} l_{AC}=(l_{x1}+\Delta l_{x1})\sin\theta_{1}\\ l_{BD}=(l_{x1}+\Delta l_{x1})\sin\theta_{3}\\ l_{AB}=l_{y1}\cdot \sin\theta_{2} \end{array}\right.$$

(17) $$\left\{\begin{array}{l} S_{d2}=l_{y1}\sin\theta_{2}\left\{\left[\left(l_{x1}+\Delta l_{x1}\right)\sin\theta_{1}+\left(l_{x1}+\Delta l_{x1}\right)\sin\theta_{3}\right]/2\right\}\\ n_{L2}=\frac{S_{d2}}{\left[S\right]} \end{array}\right.$$

where *S*_d2_ is the support area of the landing gear after tilting, and *n*_L2_ is the safety factor of the support surface of the landing gear after the tilting of the support bar.

**Figure 7:**
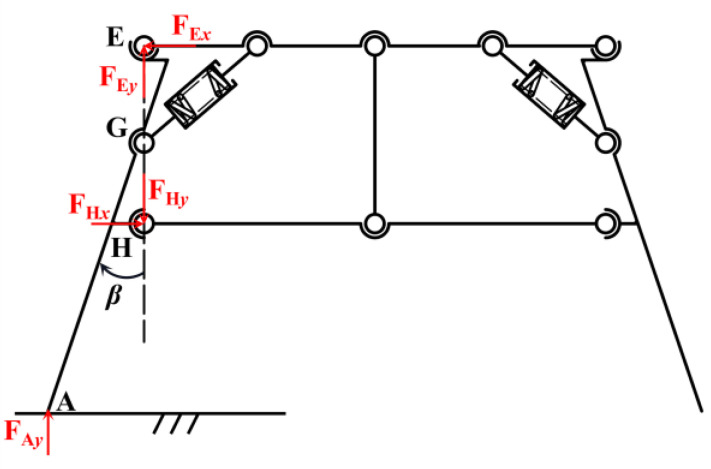
A force diagram of the support member after tilting.

The inclination of the support bars enhances the safety factor of the landing gear support surfaces. Figure 7 shows that the tilting generates *x*-direction forces *F*_EX_ and *F*_HX_ at hinge *E* and hinge *H*, respectively. The force analysis of the rod *AE* is obtained:

(18) $$\left\{\begin{array}{l} \sum F_{x}=F_{Hx}-F_{Ex}=0\\ \sum F_{y}=F_{Ay}+F_{Ey}-F_{Hy}=0 \end{array}\right.$$

The force analysis of the rod *AE* is obtained:

(19) $$\left\{\begin{array}{l} \sum F_{x}=F_{Hx}-F_{Ex}=0\\ \sum F_{y}=F_{Ay}+F_{Ey}-F_{Hy}=0 \end{array}\right.$$

The force analysis of the landing gear as a whole is obtained:

(20) $$F_{Ay}=\frac{1}{4}G+\frac{mv}{4t}$$

The torque can be found by solving for point *H*:

(21) $$F_{Ay}l_{AH}\sin\beta -F_{Ex}l_{EH}\cos\beta =0$$

The torque can be found by solving for point *A*:

(22) $$(F_{Ex}l_{AE}-F_{Hx}l_{AH})\cos\beta +(F_{Ey}l_{AE}-F_{Hy}l_{AH})\sin\beta =0$$

The above equation can be calculated by association:

(23) $$\left\{\begin{array}{l} F_{Ex}=F_{Hx}=(G+\frac{mv}{t})\frac{l_{AH}}{4l_{EH}}\tan\beta \\ F_{Ey}=(G+\frac{mv}{t})\frac{\sin\beta }{4}\\ F_{Hy}=(G+\frac{mv}{t})\frac{1+\sin\beta }{4} \end{array}\right.$$

**Figure 8:**
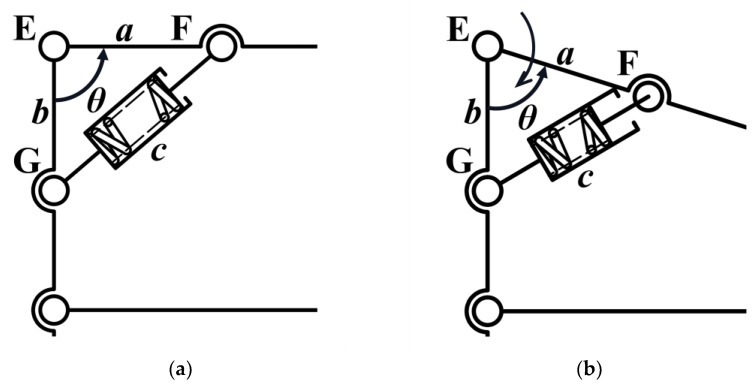
The shock absorber installation schematic. (**a**) The landing gear maintains a rectangular shape without external force. (**b**) During takeoff and landing, the landing gear is deformed by the force, and the shock absorbers cushion and dampen the vibration. (Note: *θ* is the angle between rod *EF* and rod *EG*; *c* is the distance between the two hinges of *FG*; *a* is the distance from point *E* to hinge *F*; and *b* is the distance from point *E* to hinge *G*).

The decomposition of *F*_Ay_, *F*_Hx_, *F*_Hy_, *F*_Ex_, and *F*_Ey_ into *F*_Ay1_ and *F*_Ay2_, *F*_Hx1_ and *F*_Hx2_, *F*_Hy1_ and *F*_Hy2_, *F*_Ex1_ and *F*_Ex2_, and *F*_Ey1_ and *F*_Ey2_, perpendicular and parallel to rod *AHE*, produces the force analysis of rod *AHE*, as shown in Figure 8. The force analysis of it can be obtained:

- When 0 ≤ *x* ≤ *l*_AH_, there exists a shear stress *F*_S_(*x*) and a bending moment *M*_s_(*x*), respectively:
  (24) $$\left\{\begin{array}{l} F_{s}(x)=F_{Ay1}\\ M_{s}(x)=F_{Ay1}x \end{array}\right.$$
- When *l*_AH_ ≤ *x* ≤ *l*_AE_, there exists a shear stress *F*_S_(*x*) and a bending moment *M*_s_(*x*), respectively:
  (25) $$\left\{\begin{array}{l} F_{s}(x)=F_{Ay1}-F_{Hx1}-F_{Hy1}\\ M_{s}(x)=F_{Ay1}x-(F_{Hx1}+F_{Hy1})(x-l_{AH}) \end{array}\right.$$

From the bending moment equation, the maximum bending moment of rod *AHE* at point *H* is:

(26) $$\left[M_{s}\right]=(\frac{1}{4}G+\frac{mv}{4t})l_{AH}\sin\beta$$

(27) $$n_{B}=\frac{M_{\max}}{\left[M_{S}\right]}$$

The total weight of the landing gear and the UAV is around 50 kg. The buffer period for the landing gear is *t* ≥ 0.05 s; use *t* = 0.05 s for calculations. Take *β* = 18° and [*M*_S_] = 0.164 kN∙m. The bending strength of the 2020 model aluminum profile, as per the European standard industrial selection manual, is 0.94 kN∙m. The bending safety coefficient of the support bar is *n*_B_ = 3.83, and the safety coefficient of the support surface has been enhanced by approximately 16.8%.

### 2.2. Cushion Damping Mechanism Design

According to the overall mechanism design, during the descent on a complicated landing surface, the multi-quadruple rod passive adaptive landing gear developed in this study achieves adaptability through mechanism deformation. Nevertheless, the varying sequence of the contact between each support point of the adaptable landing gear and the landing surface might easily result in a significant impact during the landing procedure. Consequently, we incorporate a cushioning and damping mechanism into the system to offer a deceleration buffer for the mechanism’s deformation. Simultaneously, a portion of the kinetic energy and gravitational potential energy during the landing process can be stored via the cushion damping mechanism. The cushion damping device maintains the overall mechanism’s form throughout flight, ensuring that after takeoff, the landing gear returns to its initial state and remains stable.

#### 2.2.1. Design Proposal

The overall landing gear mechanism consists of parallelogram mechanisms. In order to effectively slow down the deformation process of the mechanism after contacting the complex landing surface, a shock absorber is added to each four-bar mechanism to achieve a buffer damping mechanism of the landing gear. The shock absorbers must be symmetrically affixed within each parallelogram mechanism. However, if they are symmetrically positioned on opposing sides, they will be unable to function during the mechanism’s deformation. Consequently, the shock absorbers are secured to adjacent sides via hinges, with the specific mounting method shown in Figure 8. As the angle *θ* varies, the distance between the two points *F* and *G* of the two hinges changes, causing the spring in the shock absorber to deform and generate elastic force. Since the shock absorber is symmetrically installed on the parallelogram mechanism, the elastic force generated by the deformation of the mechanism will lead the mechanism to return to the rectangular condition without external force. This not only ensures that the landing gear is restored to the initial state after takeoff, but also in the process of descending, the deformation angle of the mechanism changes gradually due to the adaptation to the complex landing surface, and the elastic force in the shock absorber increases gradually, thus playing the role of damping and cushioning.

#### 2.2.2. Mounting Position Design

From Figure 8, the obstruction by the complex landing surface during the landing process leads to the change in the *θ* angle, which causes the spring deformation in the shock absorber to produce resistance:

(28) $$\left\{\begin{array}{l} c^{\prime 2}=a^{2}+b^{2}\\ c=\sqrt{a^{2}+b^{2}-2ab\cos\theta } \end{array}\right.$$

where *c*′ is the length between the two hinges of the *FG* in the rectangular state of the four-bar mechanism, and *c* is the length between the two hinges of the *FG* in the *θ*-angle state of the four-bar mechanism.

(29) $$c=f(\theta )=\sqrt{a^{2}+b^{2}-2ab\cos\theta }$$

By the fundamental inequalities:

(30) $$2ab\leq a^{2}+b^{2}$$

The equality sign of the inequality holds if, and only if, *a* = *b*. When cos*θ* ≥ 0, the compression amount of the shock absorber is the largest; when cos*θ* < 0, the stretching amount of the shock absorber is the largest. Therefore, finally, the shock absorber hinges *F* and *G* are installed at positions with equal distances from point *E* to achieve the maximum buffering effect generated by the shock absorber during the deformation of the mechanism.

### 2.3. Locking Mechanism Design

**Figure 9:**
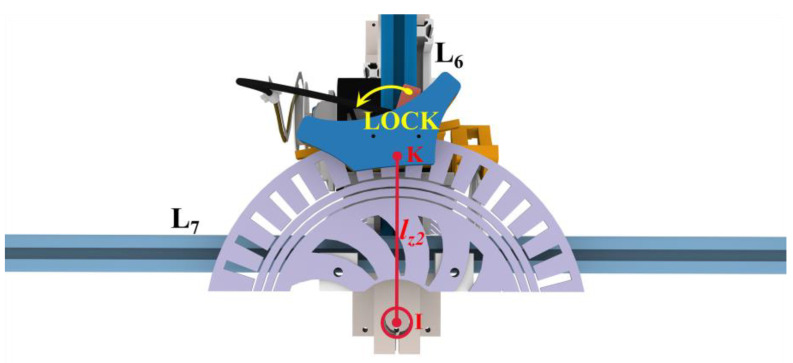
A schematic diagram of the locking mechanism.

The cushioning shock absorber mechanism generates a resistance force upon the adaptive landing gear landing on a complex surface, prompting the landing gear to revert to its rectangular shape, thereby affecting the stability of the aircraft during landing. Consequently, it is essential to lock the length of the shock absorber following the aircraft’s landing to maintain stability post-landing. Figure 9 shows that the deformation adaptation process of the mechanism involves the rotation of the *L*_7_ rod about the *L*_6_ rod. A wire-controlled disk brake component is integrated into the *L*_6_ rod. A semi-circumferential disk brake pad, compatible with the disk brake component, is incorporated into the *L*_7_ rod, which exhibits a relative motion with it. The semi-peripheral brake pads are clamped and locked by the movement of the swing arm in the wire-controlled disk brake assembly. This prevents the issue of the instability upon landing caused by the varying resistance of shock absorbers on either side. The locking mechanism clamps the brake pads after adapting to the landing surface to lock the mechanism in phase. The locking mechanism releases the brake pads at the time of takeoff. Upon release, the resisting force within the shock absorber restores the mechanism to its rectangular configuration to facilitate takeoff. The locking mechanism maintains the stability of the system post-landing by locking the phases with disk brakes. The process converts a portion of kinetic energy and gravitational potential energy into elastic potential energy, which is stored in the shock absorber during landing. During takeoff, the locking mechanism releases stored energy to realize energy reuse. The dependability of the locking mechanism is assessed by quantifying the magnitude of the braking torque generated by its friction. The disk brake system is operated by the DS5180SSG servo motor manufactured by Dongguan Dsservo Technology Co., Ltd. in China, capable of delivering a maximum torque of 90 kg∙cm. The landing gear employs a mechanical leverage ratio *η* of 5.8 for the line-controlled disk brake, with a transmission radius of *r* 0.1 m. The static friction coefficient of the disk brake is *μ* 0.4, the radius of the disk brake disk is *r*_1_ 0.2 m, and the distance between the centers of gravity *r*_2_ is 0.1 m. Subsequently, there exist:

(31) $$M_{clamp\max}=n\cdot f_{clamp\max}\cdot r_{1}=n\cdot \mu \cdot F_{clamp\max}\cdot r_{1}=n\cdot \mu \cdot \frac{T_{\max}}{r}\cdot \eta \cdot r_{1}=245.5488\;N\cdot m$$

(32) $$M_{actual}=mgn_{B}r_{2}=50\times 9.8\times 3.83\times 0.1=187.67\;N\cdot m$$

The friction of the locking mechanism provides a braking torque that is greater than the torque generated by the gravity of the landing gear landing at the safety factor condition, which is more reliable.

### 2.4. Energy Recovery System Design

**Figure 10:**
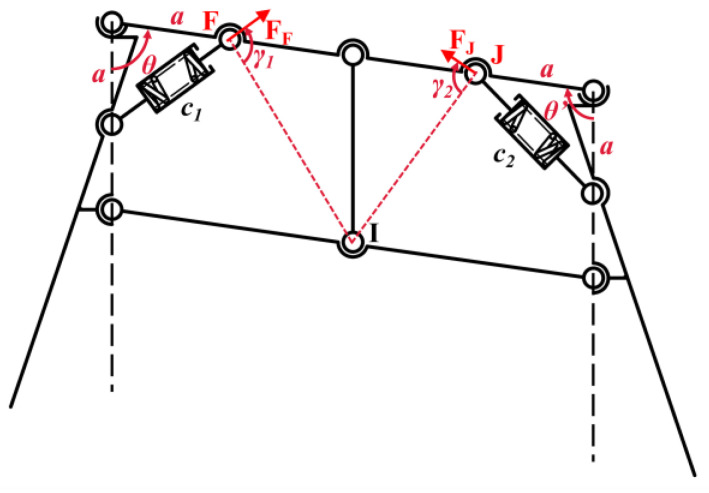
A schematic diagram of the landing gear shock absorbers during landing.

The overall mechanism design dictates that when landing on a complex surface, a portion of the kinetic and gravitational potential energy is transformed into elastic potential energy, which is stored in the shock absorber via the locking mechanism. During takeoff, the locking mechanism discharges the stored energy in the shock absorber to power the takeoff. The initial length of the shock absorber is *c*′ while the mechanism is in its undeformed state, namely in the rectangular configuration. Figure 10 shows that the lengths of the shock absorbers on either side of the mechanism post-deformation are *c*_1_ and *c*_2_. The spring in the left shock absorber is compressed, while the spring in the right shock absorber is extended.

(33) $$\theta^{\prime }=\pi -\theta$$

(34) $$\left\{\begin{array}{l} c^{\prime }=\sqrt{2a^{2}}\\ c_{1}=\sqrt{2a^{2}\left(1-\cos\theta \right)}\\ c_{2}=\sqrt{2a^{2}\left(1-\cos\theta^{\prime }\right)}=\sqrt{2a^{2}\left(1+\cos\theta \right)} \end{array}\right.$$

(35) $$\left\{\begin{array}{l} \Delta x_{1}=\sqrt{2a^{2}}-\sqrt{2a^{2}\left(1-\cos\theta \right)}\\ \Delta x_{2}=\sqrt{2a^{2}\left(1+\cos\theta \right)}-\sqrt{2a^{2}} \end{array}\right.$$

(36) $$E_{p}=\frac{1}{2}k\left[8a^{2}-4a^{2}\left(\sqrt{1-\cos\theta }+\sqrt{1+\cos\theta }\right)\right]$$

where ∆*x*_1_ is the compression of the spring in the left shock absorber, ∆*x*_2_ is the compression of the spring in the right shock absorber, and *Ep* is the energy stored in both shock absorbers.

To further increase the energy stored in the shock absorber, the spring within it is pre-compressed. The spring in the shock absorber is substituted with a spring possessing an identical *k*-value and an extended original length. So that it is already compressed before the mechanism is deformed.

(37) $$\left\{\begin{array}{l} \Delta {x_{1}}^{\prime }=\sqrt{2a^{2}}-\sqrt{2a^{2}\left(1-\cos\theta \right)}+\Delta x\\ \Delta {x_{2}}^{\prime }=\Delta x-(\sqrt{2a^{2}\left(1+\cos\theta \right)}-\sqrt{2a^{2}}) \end{array}\right.$$

(38) $${E_{p}}^{\prime }=\frac{1}{2}k\left[8a^{2}+2\Delta x^{2}+4\sqrt{2}a\Delta x-(4a^{2}+2\sqrt{2}a\Delta x)\left(\sqrt{1-\cos\theta }+\sqrt{1+\cos\theta }\right)\right]$$

(39) $$\Delta E_{p}=E_{p}\prime -E_{p}=\frac{1}{2}k\left[2\Delta x^{2}+4\sqrt{2}a\Delta x-2\sqrt{2}a\Delta x\left(\sqrt{1+\cos\theta }-\sqrt{1-1\cos\theta }\right)\right]$$

After the pre-compression of the springs in the shock absorber, the accumulated elastic potential energy is augmented by ∆*E_p_*. The pre-compression of springs before installation enhances the energy recovery. Nonetheless, the pre-compression results in an elevation of the resistance within the shock absorber following the deformation of the mechanism. The resistance in the shock absorber during pre-compression is shown in Figure 9. The locking mechanism is positioned at point I, where the resistance in the shock absorber is exerted.

(40) $$\left\{\begin{array}{l} l_{FI}=\frac{l_{z1}\sin\theta }{2\sin\gamma_{1}}\\ l_{JI}=\frac{l_{z1}\sin\theta }{2\sin\gamma_{2}} \end{array}\right.$$

(41) $$\left\{\begin{array}{l} {F_{F}}^{\prime }=k\Delta {x_{1}}^{\prime }\\ {F_{J}}^{\prime }=k\Delta {x_{2}}^{\prime } \end{array}\right.$$

(42) $${M_{I}}^{\prime }={F_{F}}^{\prime }l_{FI}\sin\gamma_{1}-{F_{J}}^{\prime }l_{JI}\sin\gamma_{2}$$

As shown in Figure 9, the distance of point *I* from the force point *K* in the brake locking position is *l*_z2_. The force of *M_I_*’ on point *K* is *FK*’. The friction force *f*_K_ and *F*_K_’ generated by the locking mechanism at point *K* are equal in value and opposite in direction. *f*_B_ is the braking force of the disk brake.

(43) $${F_{K}}^{\prime }=\frac{{M_{I}}^{\prime }}{l_{z2}}$$

(44) $$\left\{\begin{array}{l} {F_{K}}^{\prime }=\frac{l_{z1}\sin\theta }{2l_{z2}}\left[2ak\left(\sqrt{2}-\sqrt{2-2\cos\theta }\right)\right]\\ F_{K}=\frac{l_{z1}\sin\theta }{2l_{z2}}\left[2ak\left(\sqrt{2}-\sqrt{2-2\cos\theta }\right)\right]\\ \Delta E_{p}={E_{P}}^{\prime }-E_{p}\geq 0\\ {F_{K}}^{\prime }=F_{K}=f_{k}\leq F_{B} \end{array}\right.$$

Shock absorbers equipped with pre-compressed springs exhibit an identical resistance at the *K*-point after the deformation of the mechanism as they do at the original length of the spring. The shock absorber with a pre-compressed spring recuperates greater energy with an equivalent deformation of the mechanism. Consequently, the optimization utilizing pre-compressed springs enhances energy recovery. In order to verify the reliability of the energy recovery system, the ultimate load-carrying capacity of the spring damper is calculated. It is known that the pre-compression is 50% and the spring stiffness coefficient of the spring damper is 24.844 N/mm. According to the design parameters, the maximum deformation of a single shock absorber, Δ*x*_max_ = 15 mm, which corresponds to the maximum gravity at the maximum load-carrying capacity, and the safety factor is

(45) $$F_{\max}=k\cdot \Delta x_{\max}=24.844\times 15=372.66\;N$$

(46) $$F_{\mathrm{actual}}=mgn_{B}=50\times 9.8\times 3.83=1876.7\;N<nF_{\max}=6\times 372.66=2235.96\;N$$

The ultimate load capacity of the spring damper is greater than the landing gear and the gravity of the airplane landing under the safety factor condition, and the reliability is good.

## 3. Electronic Controls and Circuit Design

### 3.1. Hardware Design

The design considers the maximum of the microprocessor port use and the guideline for the stable implementation of control functions. At the same time, it needs to exhibit low power consumption, a low cost, powerful anti-interference, and other qualities. In this paper, STC32G produced by Shenzhen STC Micro Technology Co., Ltd. in Shenzhen, China (32-bit 8051 microcontroller) is chosen as the main control processor, and the appropriate circuits are selected and designed. The microprocessor core board incorporates voltage regulator chips, overcurrent protection chips, and short-circuit protection chips. This microcontroller does not require an external crystal or external reset circuit. In addition, there is a laser distance measuring module (VL53L0X produced by STMicroelectronics in Agrate Brianza, Italy), which is coupled with the microcontroller through the I2C communication protocol. The power module is a digital servo (DS5180SSG manufactured by Dongguan Dsservo Technology Co., Ltd. in Dongguan, China), and the microcontrol unit conducts the angle control via PWM. The laser range module is selected considering the qualities of a high speed, large range, low power consumption, and small size, and the VL53L0X module uses ToF technology to make its accuracy as high as ±3%. The servo selection considers that the torque must be enough to drive the locking mechanism. The selection of the DS5180SSG digital servo in the 8.4 V supply voltage can be obtained under the 0.19 s/60° response speed and a torque of more than 90 kg‧cm. Power is supplied by employing a 2 s lithium power battery; the battery volume is selected based on the premise of compactness and it can also ensure that the servo’s high current output, full power, and a maximum voltage of 8.4 V can directly drive the servo.

### 3.2. Software Design

**Figure 11:**
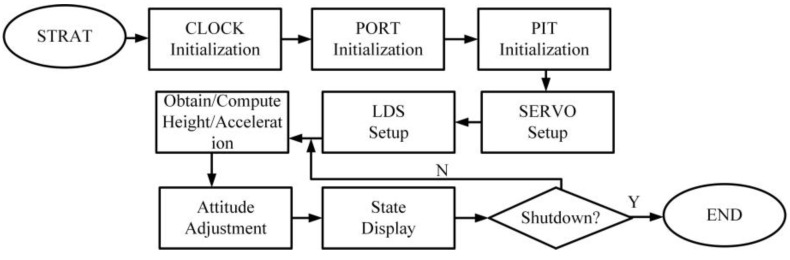
Landing gear control program flow chart.

This design employs Keil C251 developed by Advanced RISC Machines Ltd. In UK for microcontroller development, offering a comprehensive development suite that includes a C compiler, a macroassembler, a linker, library management, and a simulation debugger. Programming and debugging are conducted using the stc-isp program supplied by STC. Figure 11 shows the specific program flowchart.

**Figure 12:**
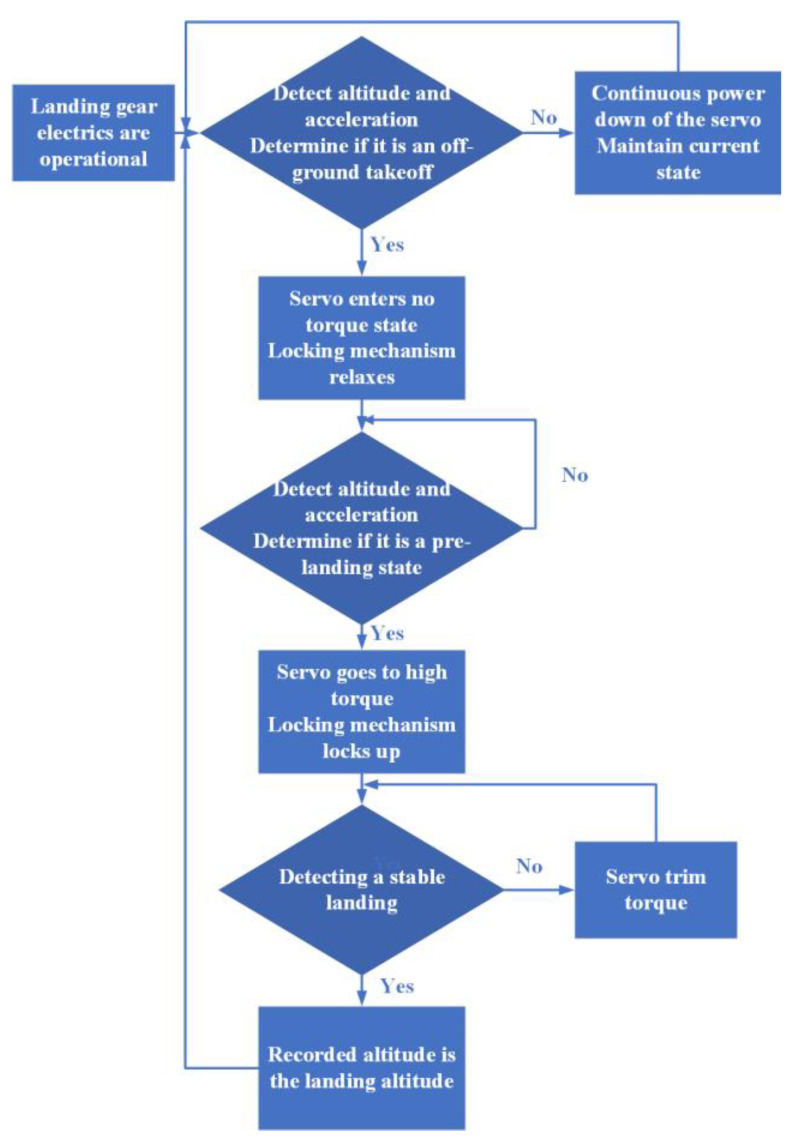
Logic block diagram of landing gear operation.

This design’s control logic employs a segmented control approach, encompassing the acquisition of height information to compute acceleration, subsequently regulating the servo to execute the corresponding tension and relaxation, thereby ensuring the program operates consistently with a high stability and minimal false triggering. Figure 12 shows the specific control logic diagram.

## 4. Experimentation and Testing

This article employs the SolidWorks 2021 software to model the complete mechanism. Aluminum profiles conforming to European standards 2020 [32] are utilized as rods in the construction of the mechanism. The mechanism’s hinge is constructed utilizing an aluminum alloy optical shaft holder equipped with flange bearings and plug bolts. The locking system uses a DS5180SSG servo with a 30 mm radius coil to pull the three-disk brakes. The average drive force allocated to each disk brake is 90 N. The semi-circumferential disk brake pads of the locking mechanism are fabricated from 4mm fiberglass plates. The servo reel, servo bracket, and pull guide are fabricated using 3D printing with PLA materials. The measurement of the assembled landing gear by electronic scales yielded a total mass of 21.32 kg. The total mass cost increased by 53.8% compared to the traditional fixed bracket structure [33].

### 4.1. Energy Recovery Experiment

Considering the inherent strength of the shock absorber, a spring with a stiffness coefficient of *k* = 24.844 was installed with 50% pre-compression. In this section, energy recovery experiments are carried out on the pre-compressed landing gear unit by measuring the lengths and angles of the two sides of the triangle where the shock absorber is located, except for the spring, and then calculating the amount of the spring compression of the shock absorber using the cosine theorem, and finally determining the energy stored in the spring. The energy recovery curves of the landing gear device in three landing states were calculated by fitting the ten sets of data collected during the landing process. The experimental setup had three different adaptations:

1. Parallel same quadrilateral bilateral barriers, i.e., only mechanism 1 or only mechanism 3 deformations;
2. Unilateral barriers, i.e., only mechanism 2 deformations;
3. Different quadrilateral bilateral barriers, i.e., only mechanisms 1 and 3 deform and have the same value of the deformation variable angle sin.

**Figure 13:**
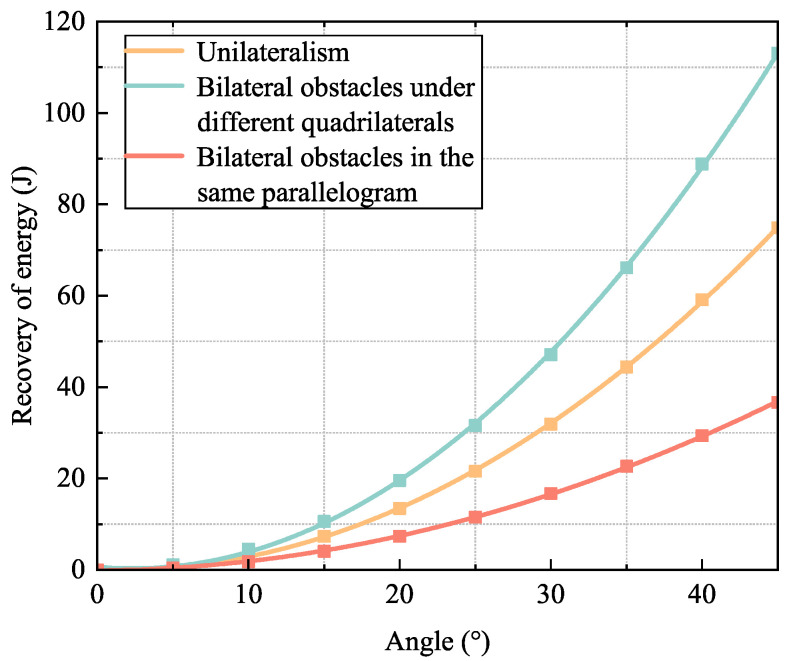
Energy recovery curves for three landing states.

Experiments utilized the deformation angle of the four-bar mechanism as the horizontal axis and the stored energy post-landing as the vertical axis. Figure 13 shows that the shock absorber equipped with pre-compressed springs exhibits a significantly higher energy storage capacity and a higher energy recovery rate. Figure 13 shows that the landing gear achieves the highest energy recovery rate of around 18.8% while encountering bilateral barriers with varying quadrilaterals, which are attributable to the simultaneous involvement of two distinct quadrilaterals in obstacle adaptation and energy recovery. In the presence of a unilateral barrier, the quadrilateral on one side of the landing gear can more effectively transform the potential energy differential into elastic potential energy, achieving an energy recovery efficiency of approximately 12.4%. Compared with the first two landing states, the bilateral obstacle of the identical quadrilateral arises from two impediments exerting force on the same parallelogram, leading to force dispersion. Consequently, a portion of the energy is expended on structural adjustments and interactions within the parallelogram, failing to be efficiently transformed into recoverable elastic potential energy, resulting in a comparatively low energy recovery rate of approximately 6.1%.

### 4.2. Actual Landing Test

**Figure 14:**
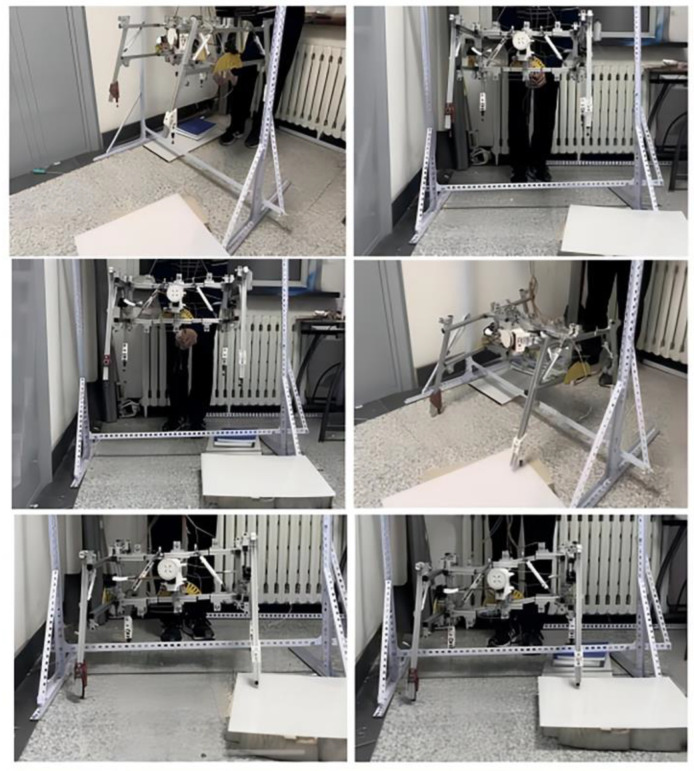
Landing gear experiment physical drawing.

**Figure 15:**
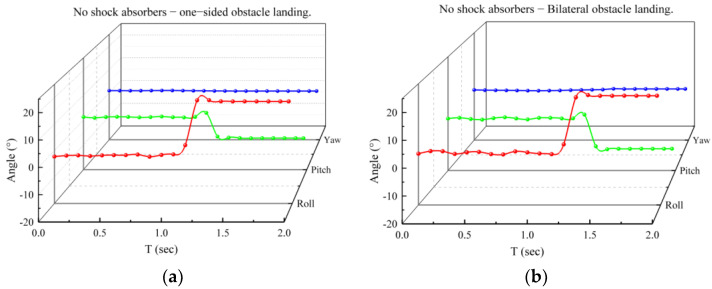

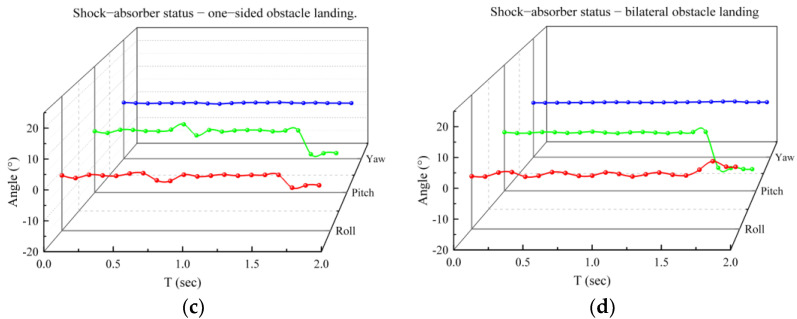
Angle of change in center of gravity during landing gear drop. (**a**) No shock absorbers, one-sided obstacle landing experiment; (**b**) no shock absorbers, bilateral obstacle landing experiment; (**c**) shock-absorbers, one-sided obstacle landing experiment; and (**d**) shock-absorbers, bilateral obstacle landing experiment.

Conducting tests with medium and large drones poses significant risks. Therefore, a gantry was constructed for this experiment, and the experiment was conducted by manually releasing the suspended landing gear. The review of the data indicates that the landing speed of UAVs and light vehicles is approximately 1 m/s. The equation *h* = (*mv*^2^)/2*g* demonstrates that the change in the center of mass from an initial speed of 0 to this speed is 0.05 m. The landing speed of the UAV’s landing gear during the test is established at 3.3 m/s, corresponding to a safety coefficient of approximately 3.3, based on the descent velocity of the UAV. The gantry construction elevates the center of mass of the landing gear to 0.5 m. A fixed pulley was employed on the gantry to alter the direction of the pull rope. The landing gear support point was elevated to a height of 0.5 m above the ground and subsequently released to simulate the landing procedure. The variation in the center of gravity during landing was assessed when the landing gear contacted unilateral and bilateral impediments, both with and without shock absorbers, as seen in Figure 14. The angular velocity and angular acceleration of the center of gravity during the descent of the landing gear were monitored in real time using a gyroscope and subsequently transformed into real-time angular variations. In the state devoid of a shock absorber, as illustrated in Figure 15a,b, the angular variation in the center of gravity remains consistently unstable during the descent of the landing gear and the base obstacle maneuver, resulting in a diminished overall stability. The measurement results depicted in Figure 15c,d indicate that, with the shock absorber installed, the landing gear effectively stabilizes the center of gravity’s angle during descent and upon contact with obstacles, facilitating a stable landing. The angular variation in all directions ranges from −10° to 25°.

## 5. Conclusions

This paper designs and tests an innovative adaptive landing gear aimed at improving the safety and adaptability of UAV landings in complex terrain. Utilizing multiple mutually perpendicular four-bar mechanisms and a locking system with a single laser rangefinder sensor, this paper attains the passive adaptation of the landing gear to complex obstacle surfaces, significantly reducing the control complexity and the structural intricacy of the system. The testing findings demonstrate that the adaptive landing gear can effectively accommodate landing surfaces with differing height differentials while also displaying a superior damping effect and center of gravity stability throughout the landing procedure, hence guaranteeing the UAV’s safe landing. The addition of a buffer–damping system and a locking mechanism enhances the stability and energy recovery efficiency of the landing gear. The shock absorber design of the pre-compressed spring enhances the resistance after deformation and achieves an energy recovery efficiency of 18.8%, thereby providing additional energy support for the takeoff of the UAV. This research presents an innovative viewpoint on UAV landing gear design and lays the groundwork for future UAV applications in several domains. The continuous progression of UAV technology indicates that the development of adaptive landing gear will substantially lower maintenance expenses and expand the range of UAV applications. The existing design continues to exhibit issues, including the potential of attitude control dispersion and an excessive system weight. Future research will concentrate on the lightweight optimization of the mechanism while simultaneously integrating a microcontroller to monitor and regulate the real-time release timing of the disk brakes, thereby ensuring phase consistency in the energy release process and enhancing the system’s stability and reliability.

## Data Availability

Data are contained within the article.
